# Fas-Mediated Apoptosis Regulates the Composition of Peripheral αβ T Cell Repertoire by Constitutively Purging Out Double Negative T Cells

**DOI:** 10.1371/journal.pone.0003465

**Published:** 2008-10-21

**Authors:** Abdiaziz S. Mohamood, Dylan Bargatze, Zuoxiang Xiao, Chunfa Jie, Hideo Yagita, Dawn Ruben, Julie Watson, Shukti Chakravarti, Jonathan P. Schneck, Abdel Rahim A. Hamad

**Affiliations:** 1 Department of Pathology, Johns Hopkins University School of Medicine, Baltimore, Maryland, United States of America; 2 Institute of Genetic Medicine, Johns Hopkins University School of Medicine, Baltimore, Maryland, United States of America; 3 Department of Molecular and Comparative Pathobiology, Johns Hopkins University School of Medicine, Baltimore, Maryland, United States of America; 4 Department of immunology, Juntendo University School of Medicine, Tokyo, Japan; 5 Department of Medicine, Johns Hopkins University School of Medicine, Baltimore, Maryland, United States of America; University Paris Sud, France

## Abstract

**Background:**

The Fas pathway is a major regulator of T cell homeostasis, however, the T cell population that is controlled by the Fas pathway in vivo is poorly defined. Although CD4 and CD8 single positive (SP) T cells are the two major T cell subsets in the periphery of wild type mice, the repertoire of mice bearing loss-of-function mutation in either Fas (lpr mice) or Fas ligand (gld mice) is predominated by CD4^−^CD8^−^ double negative αβ T cells that also express B220 and generally referred to as B220^+^DN T cells. Despite extensive analysis, the basis of B220^+^DN T cell lymphoproliferation remains poorly understood. In this study we re-examined the issue of why T cell lymphoproliferation caused by gld mutation is predominated by B220^+^DN T cells.

**Methodology and Principal Findings:**

We combined the following approaches to study this question: Gene transcript profiling, BrdU labeling, and apoptosis assays. Our results show that B220^+^DN T cells are proliferating and dying at exceptionally high rates than SP T cells in the steady state. The high proliferation rate is restricted to B220+DN T cells found in the gut epithelium whereas the high apoptosis rate occurred both in the gut epithelium and periphery. However, only in the periphery, apoptosis of B220^+^DN T cell is Fas-dependent. When the Fas pathway is genetically impaired, apoptosis of peripheral B220^+^DN T cells was reduced to a baseline level similar to that of SP T cells. Under these conditions of normalized apoptosis, B220^+^DN T cells progressively accumulate in the periphery, eventually resulting in B220^+^DN T cell lymphoproliferation.

**Conclusions/Significance:**

The Fas pathway plays a critical role in regulating the tissue distribution of DN T cells through targeting and elimination of DN T cells from the periphery in the steady state. The results provide new insight into pathogenesis of DN T cell lymphoproliferation.

## Introduction

The Fas receptor is the prototypical member of the tumor necrosis factor receptor (TNFR) family of cytokines and is constitutively expressed on T cells [1]–[3]. The Fas ligand (FasL) is a member of the tumor necrosis factor (TNF) family and its expression is tightly regulated and induced after TCR activation [1]–[3]. Engagement of Fas by FasL leads to recruitment of Fas-associated death domain (FADD) and activation of the caspase cascade causing cell death by cleavage of molecules that regulate cellular structure and integrity [2], [3]. In vitro studies of Fas-mediated apoptosis using hybridomas and primary T cells established the paradigm of Fas-mediated activation-induced cell death (AICD) as a major regulator of T cell clonal expansion [4]–[6]. The in vivo role of the Fas pathway, however, is poorly understood; whereas some studies reported a delay or defect in deletion of Fas-deficient T cells in response to foreign antigen stimulation [7], [8], several other studies showed that antigen-activated T cells undergo apoptosis in vivo in the absence of a functional Fas pathway [9]–[12]. Furthermore, there is little, if any, defect in thymic negative selection in the absence of functional Fas pathway [13]–[16]. Yet, massive numbers of a peculiar type of TCRαβ cells that is referred to as double negative (DN) T cells due to the lack of CD4 and CD8 coreceptors, gradually accumulate in the lymph nodes and spleens of mice with loss-of-function mutation in Fas (lpr) or Fas ligand (gld) leading to qualitative changes in the composition of peripheral T cell repertoire and to DN T cell lymphoproliferation [17]–[19]. These DN T cells are positive for B220, an isoform of CD45 molecule that is normally expressed by B cells and hence are generally referred to as B220^+^ DN cells [17]. Normal peripheral CD4 and CD8 T cells do not normally expressed B220 but it is expressed on activated T cells undergoing apoptosis following injection of mice with staphylococcal enterotoxin B superantigen [20]–[22]. Phenotypically similar B220^+^ DN T cell population causes lymphoproliferation children bearing mutations in Fas, FasL or caspase 10 [23], [24]. However, the basis of B220^+^ DN T cell lymphoproliferation as a function of impaired Fas pathway remains poorly understood.

Although several genetic deficiencies lead to T cell lymphoproliferation, as in scurfy mice that lack functional Foxp3 [25], [26] or in CLTA-4 deficient mice [27], only the lymphoproliferation caused by impairment of the Fas pathway is dominated by DN T cells [17]. B220^+^ DN T cells are angeric [28] and are not actively proliferating in the lymph nodes and spleen of mutant mice but were reported to be proliferating in the liver of MRL/lpr mice [29]. The lymphoaccumulation of B220^+^ DN T cells is severely reduced in mice lacking MHC class 1 [30], [31] or treated with anti-CD8 mAb [32] suggesting the majority of DN T cells develop from CD8^+^ thymic precursors selected by interaction with class I MHC molecules. Furthermore, CD8 gene is demethlyated in B220+ DN T cells indicating prior expression of CD8 coreceptor and perhaps a passage through CD4^+^8^+^ thymic stage of differentiation [33]. However, in vivo treatment of lpr and gld mice with SEB did not lead to conversion of CD8 T cells into B220^+^ DN T cells [31]. However, B220^+^ DN T cells were reported to exist in the appendix of wild type mice and hence their development may be independent of lpr or gld mutation [34].

In this study, we show that naturally existing DN T cells are proliferating and dying at exceptionally high rates than SP T cells under the physiological steady state conditions. The high proliferation rate is restricted to DN T cells found in the gut epithelium whereas the high apoptosis rate occurred both in the gut epithelium and peripheral lymphoid tissues. However, only in the periphery, DN T cell apoptosis is Fas-dependent. The high sensitivity of DN T cells to Fas-mediated apoptosis in the periphery appears to be an important factor in maintaining their polarized tissue distribution and prevention of their accumulation in the periphery of wt mice. These results have important implications for understanding pathogenesis of B220+ DN T cell lymphoproliferation.

## Results

### Acutely activated SP T cells are not a major source of DN T cells in FasL-deficient gld mice

**Figure 1:**
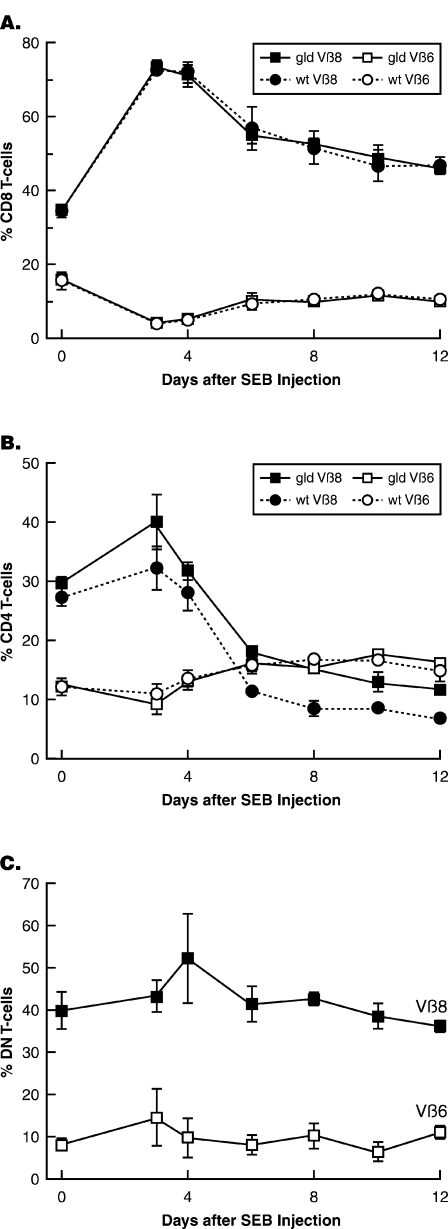
SEB activation of gld CD8 or CD4 T cells in vivo does not lead to their conversion into DN T cells. C3H-gld mice (14-week-old) or their age-matched C3H-wt mice were immunized i.p. with SEB (100 µg/mouse). Frequencies of SEB-reactive Vβ8^+^ T cells among the CD8 (A), CD4 (B), and DN (C) subsets in peripheral blood were determined on the indicated days. Similar results were obtained in lymph nodes and spleens when the experiment is terminated. Frequencies of Vβ6^+^ T cells were used as negative controls. Results show mean±SD from three mice per group.

DN T cells gradually accumulate in periphery of mice bearing lpr mutation of Fas receptor or gld mutation of FasL leading to T cell lymphoproliferation. However, there is little or no effect of lpr and gld mutations on expansion and contraction of exogenously activated SP T cells [9]–[11] indicating that the death of acutely activated T cells is largely Fas-independent. To rule out the possibility that some activated SP T cells could have downregulated their coreceptors and persisted as DN T cells, we analyzed whether superantigen activation of Vβ8-bearing SP T cells leads to long-term increase in Vβ8-bearing DN T cells. Unlike in most previous studies [9]–[12], but similar to the study by Giese et al [31], we did our analysis in 14-week-old gld mice that harbored significant numbers of DN T cells in their peripheral repertoire. We reasoned that the in vivo conditions at this age might favor SP into B220^+^ DN T cell conversion, if it ever to happen. We injected mice with staphylococcal enterotoxin B (SEB) superantigen, which stimulates T cells in a Vβ8 specific fashion and assessed its impact on the percentages of Vβ8-bearing DN and SP T cells. Vβ8^+^ T cells constitute about 30% of murine peripheral SP T cells and they expand to about 45% of T cells within 72 h after SEB stimulation followed by deletion [35]. We predicted that the frequency of Vβ8^+^ subset within the B220^+^ DN T cell population would increase and remained significantly high if activated Vβ8^+^ SP T cells were converted into long-lived B220^+^ DN T cells. Vβ6^+^ T cells, which are not activated by SEB, were used as internal controls. In accordance with previous studies [9]–[11], Vβ8^+^CD8^+^ T cells expanded and disappeared with similar kinetics and potency in gld and wt mice **(** **Fig. 1A** **)**. Similarly, there was no significant difference in the expansion and contraction of Vβ8^+^CD4^+^ T cells in gld and wt mice although deletion was slightly delayed in gld mice **(** **Fig. 1B** **)**. Vβ8^+^ B220^+^ DN T cells, which constituted about 40% of B220^+^ DN T cells in gld mice slightly expanded and rapidly contracted in response to SEB activation **(** **Fig. 1C** **)**. If anything, the frequency of Vβ8^+^DN T cells at the end of the analysis period was lower than their original frequency. These results extended a previous study showing that stimulation with SEB does not accelerate B220^+^ DN T cell accumulation [31]. Vβ8^+^ B220+ DN T cells in the periphery of wt mice maintained their very low frequency throughout the experiment and thus, as expected, were not significantly influenced by superantigen stimulation (data not shown). We concluded that B220^+^ DN T cell homeostasis is not significantly affected by exogenous antigen activation of SP T cells.

### Significant differences between the gene expression profiles of gld B220^+^ DN and SP T cells

**Figure 2:**
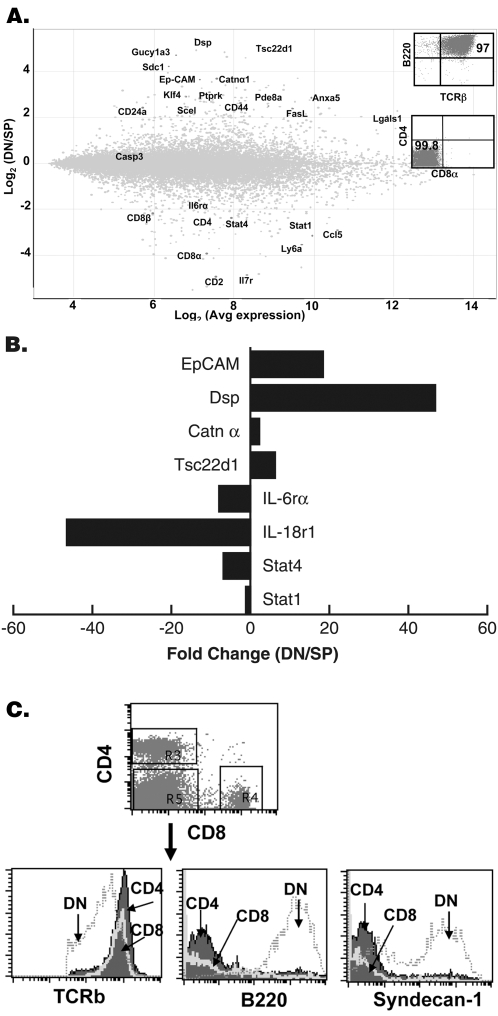
DN T cells have a unique transcript profile. (A) Comparison of the transcript profiles of DN T cells and autologous SP T cells (combined CD4 and CD8 T cells) isolated from inguinal and axillary lymph nodes of gld mice as described in Materials and Methods. The insets show purity and hallmarks properties of DN T cells including expression of TCR and B220 and lack of CD4 and CD8 on their surface. The Minus versus Average (MvA) plot shows intensity log-ratio M = log_2_ (DN/SP) versus mean log-intensity A = [(log_2_ (DN)+log_2_ (SP)]/2. Out of 32,000 transcripts examined, expression of 160 genes was at least 3-fold higher in DN than in SP T cells. Highly expressed genes including junctional adhesion molecules, hydrolyase enzymes, and apoptotic death molecules are highlighted. Selected under-expressed genes in DN T cells relative to SP T cells are also highlighted. (B) Validation of expression of selected genes by real-time PCR. Expression level of each gene was normalized relative to expression of 18 s rRNA in the same cell subset. X-axis shows -fold change in expression of indicated genes in DN T cells relative to SP T cells. (C) Validation of specific expression of sdc1 by flow cytometry. Splenocytes were isolated from 16-week-old C3H-gld/gld mice, stained with APC-TCRβ, PerCP-CD4, FITC-CD8α, and PE-sdc1 or with APC-TCRβ, PerCP-CD4, FITC-CD8α, and PE-B220 mAbs and analyzed by FACS. Dot plot: TCR^+^ cells were gated followed by specific gating of DN (R5), CD4^+^ (R3), and CD8^+^ (R4) subsets. Histograms: Overlays show relative expression of TCR, B220, and sdc1 by gated CD4, CD8, and DN subsets.

The gene profiling was used to determine whether gld B220^+^ DN T cells were different from SP T cells at the transcript level. We analyzed the transcript profile of a highly purified B220^+^ DN and SP T cell populations **(** **Fig. 2A, insets** **)** using Affymetrix Mouse Genome 430 arrays. We identified genes that were differentially expressed in B220^+^ DN T cells relative to conventional SP T cells as described in Materials and Methods. Validity of the comparison was indicated by the detection of genes encoding proteins normally expressed by SP but not by B220^+^ DN T cells **(** **Fig. 2A** **)**. These included genes for CD4, CD8α, CD8β, and CD2 molecules. Surprisingly, however, the top-scoring genes in B220^+^ DN T cells encoded molecules normally expressed by intestinal epithelia but not by conventional αβT cells. Syndecan-1 (sdc1), a primary surface marker for intestinal epithelia [36], and epithelial cell adhesion molecule (Ep-CAM; previously called Tacstd1), a regulator of homophilic interactions between epithelia [37], were highly overexpressed in B220^+^ DN T cells. In addition, desmoplakin (DSP) [38], [39]; catenin α [40], and galectin 1 [(Lgals1) [41]] that regulate junctional interactions were differentially expressed by B220^+^ DN T cells. Genes for hydrolyase and lyase enzymes (PtPRK, Pde8α, and Gucy1a3) that degrade the extracellular matrix were also differentially expressed in B220^+^ DN T cells. Importantly, B220^+^ DN and SP T cells were isolated from peripheral lymph nodes (inguinal and axillary) of gld mice, thereby excluding the possibility of contamination of microarray samples with intestinal epithelial cells. Furthermore, expression of sdc1 and junctional adhesion genes by gld B220^+^ DN but not gld SP T cells indicated that their expression was specific and was not due to a general defect in T cells caused by gld mutation. We confirmed sdc-1 expression by peripheral B220^+^ DN T cells at the protein level by using flow cytometry **(** **Fig. 2C** **)**. In addition, we used quantitative real-time PCR to confirm expression of a set of selected genes **(** **Fig. 2B** **)**. Thus, B220^+^ DN T cells in gld mice have an unconventional transcript profile. Whether this profile is acquired as result of complex differentiation process from mature SP T cells or represents a unique lineage is currently unknown.

### DN T cells naturally residing in the gut epithelium expressed epithelial cell marker sdc1

**Figure 3:**
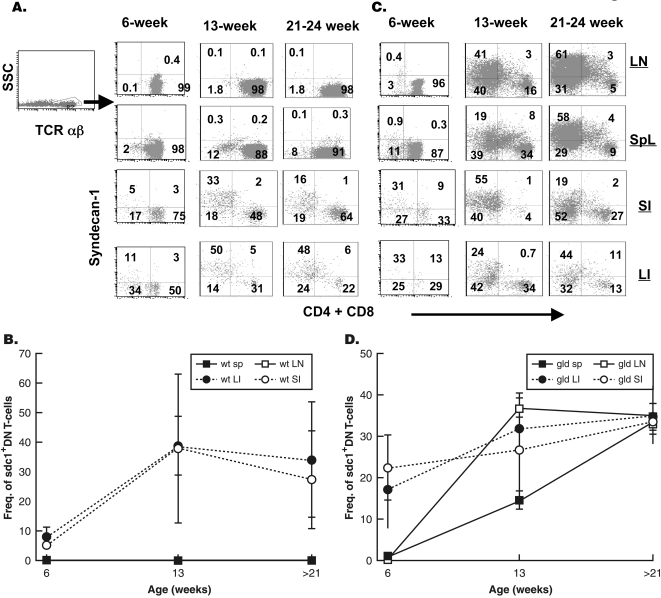
Sdc1^+^ B220+ DN T cells accumulate in an age-dependent manner in the gut epithelium of wt mice with intact Fas pathway. T cells were isolated from the periphery and gut epithelium of wt or gld C3H mice of different ages as described in Materials and Methods. Isolated cells from lymph nodes (LN), spleen, small intestine (SI), and large intestines were stained with APC-TCRβ, PerCP-CD4, PerCP-CD8α, anti-B220 and PE-sdc1 and analyzed by FACS. CD4 and CD8 T cells were included in one subset (CD4 and CD8) by simultaneously staining samples with PerCP-conjugated anti-CD4 and PerCP-conjugated anti-CD8 mAbs. This allowed us to compare directly SP (CD4^+^ and CD8^+^) and DN (CD4^−^CD8^−^) TCR^+^ subsets. Frequencies of sdc1^+^ DN T cells in the periphery and gut epithelium of wt (A) and gld (C) mice are shown. Numbers indicate the percentage of positive cells in each quadrant. Sdc1^+^ cells in the upper right quadrants in the lymph nodes and spleen of 13- and 21- to 24-week-old gld mice were CD4 T cells (not shown). Percentages of sdc1^+^ DN T cells relative to total T cells in the periphery and gut epithelium of wt (B) and gld (D) mice are shown. DN T cells isolated from the gut epithelium expressed B220 (Fig. 4A and data not shown). Results are expressed as mean±SD from three independent experiments with two to three mice per group. LN, Lymph nodes, SI, small intestine, Li, large intestine.

**Figure 4:**
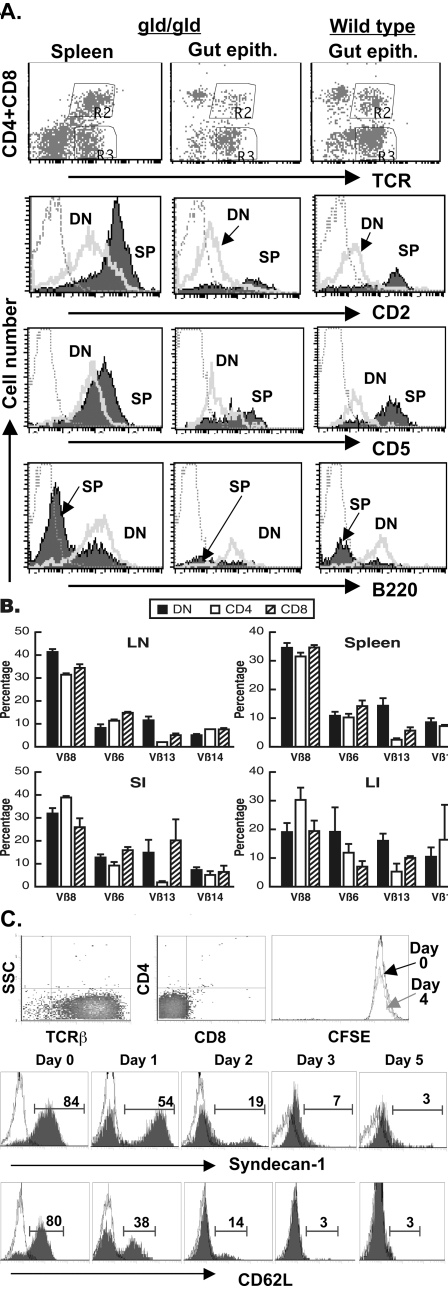
Phenotypic similarities of peripheral and intestinal DN T cells. (A) Single cell suspensions from spleens of gld mice or intestine (small and large intestines were combined) of gld or wt mice were stained for APC-TCRβ, PerCP-CD4, and PerCP-CD8α, and FITC-CD2, CD5 or B220. TCR^+^ cells were gated and expression of indicated molecules by DN and SP subsets were determined. Spleens of wt mice are not included in the analysis because of the paucity of DN T cells. (B) Similar distribution of Vβ expression by DN T cells. Peripheral T cells and IEL were isolated from 12-week-old C3H-gld/gld mice and surface-stained for TCR, CD4, CD8, and Vβ6, Vβ8, Vβ13 or Vβ14. After gating on TCR^+^ cells, the frequencies of different Vβs among DN, CD4 or CD8 T cells were determined. Data show mean±SD from one of two independent experiments. (C) Downregulation of sdc1 by TCR activation. Top panel: Analysis of TCR (left dot plot); and CD4 and CD8α expression (right dot plot) by freshly isolated DN cells prior to culture. The isolated cells were stained with TCR, CD4 and CD8 specific antibodies and their purity assessed by flow cytometry (>95% of isolated cells expressed TCR (left dot plot) and lacked CD4 and CD8 expression (middle dot plot). The histogram shows CFSE intensity in DN T cells before (day 0) and after (day 4) TCR activation. Bottom panel: Kinetics of sdc1 and CD62L downregulation by DN T cells in response to CD3/CD28 stimulation. Data from one of two independent experiments are shown.

In normal animals, B220+ DN T cells are rare in the periphery but abundant in the gut epithelium. Therefore, we determined whether sdc1 is expressed by intraepithelial TCRαβ^+^ DN subset. We particularly analyzed the gut epithelium for T cells bearing a similar phenotype (i.e. TCRαβ^+^, sdc1^+^, B220^+^, CD4^−^, and CD8^−^, CD2^−^). There was a small population of sdc1-expressing αβT cells in the epithelium of large intestine and to a lesser degree of the small intestine of 6-week-old wt mice **(** **Fig. 3A and B** **)**. The frequency of sdc1^+^ DN T cells increased with age in both the small and large intestines until they became the major αβT cell population in the large intestine of 21- to 24-week-old wt mice. They remained, however, confined to the gut epithelium as very few sdc1^+^ DN T cells were detected in the periphery of wt mice. The DN T cells in the gut epithelium of wt mice also expressed B220 but lacked CD2 (data not shown and see Fig. 4A). Sdc1-expressing DN T cells with similar patterns of accumulation were detected in the gut epithelium of nonobese diabetic (NOD) mice indicating that they were not limited to C3H mice (data not shown).

### Intact Fas pathway is required for confining sdc1-expressing DN T cells to the gut epithelium

In the absence of functional Fas pathway, sdc1-expressing B220+ DN T cells were simultaneously detected in the periphery and gut epithelium of gld mice **(** **Fig. 3C and D** **)**. Parallel accumulation of sdc1- expressing DN T cells in the periphery and gut epithelium was also seen in Fas-deficient C3H-lpr mice indicating that it caused by impairment of either Fas receptor or its ligand (data not shown). Impairment of the Fas pathway also broke down compartmentalization in NOD mice bearing gld mutation indicating the generality of our findings (data not shown). As discussed below, while the majority of DN T cells that accumulated in the periphery of gld mice expressed sdc1, some DN T cells expressed low or no sdc1.

The majority of DN T cells in the periphery of gld mice shared key surface characteristics (CD4^−^, CD8^−^, CD2^−^, CD5^−^, sdc1^+^, B220^+^, CD24A^+/−^, Thy1^+/−^) with DN T cells in the gut epithelium. The gld mutation did not influence the phenotype of DN T cells in the gut epithelium, as the surface profiles of DN T cells in the gut of wt and gld mice were comparable **(** **Fig. 4A, middle and right panels** **)**. Due to paucity of DN T cells in the periphery of wt mice we were not able to compare their phenotype to that of gut epithelium. The spectrum of TCR-Vβ expression by DN T cells in the periphery and gut epithelium was also similar **(** **Fig. 4A and B** **and data not shown)**. However, a higher proportion of DN T cells in the periphery than in the gut epithelium of gld mice expressed sdc1. But after TCR activation, DN T cells progressively lost sdc1 in a time-dependent pattern that mirrored the pattern of CD62L downregulation. Dominance of sdc1^−/low^ DN T cells of the activated culture was not due to an outgrowth of a few contaminating sdc1^−/low^ DN T cells as gld DN T cells were anergic in vitro [42] and did not proliferate in response TCR stimulation **(** **Fig. 4C** **)**. Because of difficulty in isolating a pure sdc1^+^ DN T cell population from the gut epithelium, this experiment was performed on peripheral sdc1^+^ DN T cell population. Thus, expression of sdc1 by DN T cells is dynamic and negatively regulated by TCR signals.

### DN T cells are chronically proliferating in the gut epithelium but not the periphery regardless of gld mutation

**Figure 5:**
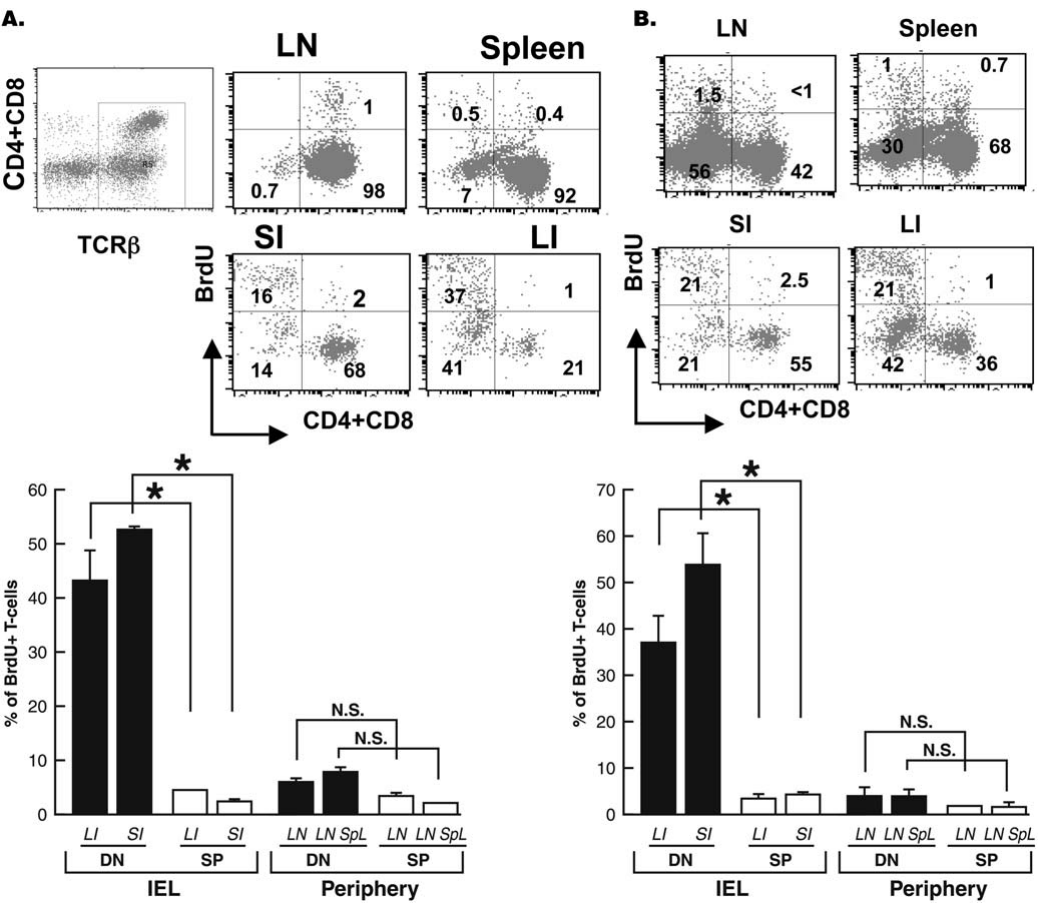
Rapid proliferation of intestinal but not peripheral DN T cells in the steady state. Wild-type and gld mice (12-week-old) received two i.p. injections of BrdU during a 24-h period as described in Materials and Methods. Peripheral T cells and IEL were isolated and stained with APC-TCRβ, PerCP-CD4/PerCP-CD8α, FITC-BrdU and PE- sdc1 specific antibodies. TCR^+^ cells were gated and percentage of BrdU^high^ in the DN or SP (CD4^+^+CD8^+^) subsets in wt (A) and gld (B) mice were determined. The graph below each panel shows mean±SEM from two independent experiments with 2–3 mice per each genotype. *, P<0.05; N.S., not significantly different.

To further compare DN and SP T cells, we assessed their in vivo proliferation in the steady state. We found that DN T cells in the gut epithelium were rapidly dividing with approximately 50% in the small intestine of wt mice incorporating BrdU within a 24-h period **(** **Fig. 5** **)**. DN T cells in the large intestine were also dividing potently but at a slightly lower rate than that in the small intestine **(** **Fig. 5A** **)**. Rapid proliferation was a unique feature of DN αβT cells in the gut epithelium as less than 5% of SP αβT cells proliferating in the 24-h period. Similar results were obtained after 8 days of continuous BrdU administration (data not shown). In contrast, DN T cells in the periphery of wt mice were not rapidly proliferating but cycling at comparable rate as SP T cells. The gld mutation did not influence the high proliferation rate of DN T cells in the gut epithelium, as levels of BrdU uptake by DN T cells in the gut of wt and gld mice were comparable **(** **Fig. 5B** **)**. BrdU^+^ DN T cells were found in both sdc1^−^ and sdc1^+^ subsets (data not shown) suggesting that proliferation is a characteristic of all DN T cells in the gut epithelium. In accordance with previous studies [43], DN T cells in the periphery of gld mice were not actively proliferating as they were cycling at a rate that was slightly but not significantly higher than that of SP T cells. A similarly high proliferation rate of DN T cells was detected in the gut epithelium of NOD mice bearing wt or mutant FasL indicating generality of our findings (data not shown). These results show that DN, unlike SP, αβT cells were uniquely proliferating at an intense rate in the gut epithelium regardless of whether the host is FasL-deficient or sufficient.

### DN T cells are selectively eliminated from the periphery of wild type mice by Fas-mediated apoptosis

**Figure 6:**
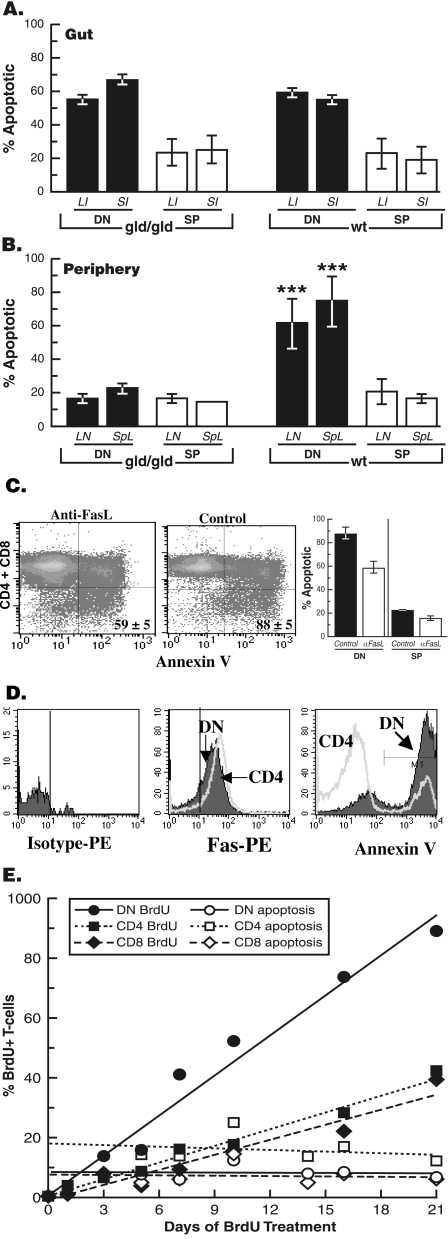
Fas-mediated apoptosis eliminates DN T cells from the periphery but not the gut epithelium. (A) Apoptosis of DN T cells in the gut epithelium is Fas-independent. IEL from 12-14-week-old C3H-gld and wt mice were stained for TCR, CD4/CD8, and annexin V binding. The graph show percentages of annexin V^+^, TCR-gated DN or SP in small (SI) and large intestine (LI) and of gld/gld and wt mice. Results are presented as mean±SEM (n = 4). There is no significant difference in apoptosis of DN T cells or SP T cells between wt and gld mice; p>0.05). (B) Apoptosis of peripheral DN T cells is Fas-dependent. Lymph node and spleen cells were stained for TCR, CD4/CD8, and annexin V binding. The frequencies of annexin V^+^ DN T cells were determined as above. The graph shows apoptosis of gated DN and SP T cells in lymph nodes (LN) and spleens of gld/gld and wt mice. The results are presented as average±SEM (n = 6 per group). Apoptosis of peripheral DN T cells is significantly higher (***, *p*<0.0001) than that of SP T cells in wt mice but in the absence of functional FasL in gld mice is comparable to that of SP T cells. (C) Wild-type C3H mice were injected i.p. with anti-FasL (0.5 mg) or control saline twice within a 24-h period. Lymph node and/or spleen cells from mice in each group were stained for TCR, CD4+CD8, and annexin V and analyzed by FACS. TCR+ cells were gated and analyzed for annexin V binding by SP (CD4^+^/CD8^+^) and DN (CD4^−^/CD8^−^) subsets. Quadrants were set using samples stained with TCR, CD4/CD8 but not annexin V. The data are pooled from two independent experiments (n = 4); numbers indicate mean±SEM of annexin V^+^ DN T cells. The graph shows mean±SEM of apoptosis. Apoptosis of DN T cells was significantly reduced by FasL blockade; p<0.0376. Apoptosis of SP T cells, which is significantly lower than of DN T cells, was further reduced by FasL blockade from 23±2 to 16±0.2; p<0.042. (D) DN T cells that accumulate in the periphery of gld mice are sensitive to FasL-induced apoptosis in the absence of concomitant TCR stimulation. DN and CD4 T cells were isolated from lymph nodes of gld mice and assessed for purity as described in Material and Methods (each >95% pure, data not shown). Levels of Fas expression by DN and CD4 T cells isolated from C3H-gld/gld mice were determined (middle histogram). Left histogram shows isotype control staining. Right histogram shows apoptosis of DN (80%) and CD4 (19%) T cells incubated overnight with soluble FasL in the presence of a crosslinker without TCR activation. Background staining of DN T cells incubated with the enhancer was less than 20%. Results from one of two independent experiments are shown. (E) Higher rate of BrdU^+^ DN T cell accumulation in the periphery of gld mice. C3H-gld/gld mice were injected i.p. with 2 mg of BrdU and then maintained on BrdU (0.8 mg/ml) in drinking water for 21 days. Rates of BrdU labeling and apoptosis of DN and SP T cells were monitored in pooled peripheral blood from mice in the same group (n = 3 per group). The best-fit lines represent the relative rate of accumulation of different subsets.

High proliferation rate must be met with reciprocally high apoptosis rate for maintenance of normal cellular homeostasis. We therefore determined whether DN T cells were also dying at higher rate than SP T cells in the steady state. We found that DN T cells were indeed undergoing higher apoptosis rate than SP T cells both in the gut epithelium and secondary lymphoid organs using Fas-independent and Fas-dependent mechanisms, respectively. Apoptosis of DN T cells in the gut epithelium was Fas-independent as DN T cells died at comparable rates in the gut epithelium of wt and gld mice **(** **Fig. 6A** **)**. Consequently, there was minimal or no impact of gld mutation on the frequency of DN T cells in the gut epithelium (see Fig. 3). These results corroborate previous findings that the Fas pathway plays no major role in apoptosis of IEL [44]. In contrast, apoptosis of DN T cells in the periphery was Fas-dependent. DN T cells were dying at significantly higher rate than SP T cells in the periphery of wt mice **(** **Fig. 6B** **)**. The gld mutation significantly reduced apoptosis of DN T cells and normalized it to a level similar to that of SP T cells. Blockade of the Fas pathway by using FasL-neutralizing (MFL4) antibody [45] significantly inhibited DN T cell apoptosis in the periphery of wt mice confirming that it was Fas-dependent **(** **Fig. 6C** **)**. Continued antibody blockade of FasL led to increased percentage of DN T cells in the periphery of wt mice as recently reported by our lab [46] and by another group [47]. Furthermore, DN T cells that accumulated in the periphery of gld mice had been primed for Fas-mediated apoptosis as they died when exposed to recombinant FasL in the absence of concomitant TCR activation **(** **Fig. 6C** **)**. Preferential elimination of B220^+^ DN T cells has also been reported when peripheral lymphocytes from gld mice were exposed to bioactive FasL-bearing vesicles [48]. These results indicate that Fas-mediated apoptosis plays a critical role in clearing DN T cells from the periphery.

Finally, we simultaneously monitored proliferation, turnover, and apoptosis of gld B220+ DN, CD4, and CD8 T cells in the peripheral blood of gld mice over a 21-d period using the BrdU and annexin V assays **(** **Fig. 6E** **)**. Mutant mice were injected with BrdU and then maintained on drinking water containing BrdU. There were comparable levels of labeling of T cell subsets 3 and 24 h after BrdU administration, confirming similar cycling rates indicated above (**Fig 5B** **)**. The apoptosis rate of DN T cells in the absence of functional Fas pathway was comparable to that of CD8 T cells at any given time during the 21-d period and was slightly lower than that of CD4 T cells. Yet, the frequency of BrdU^+^ DN T cells in peripheral blood steadily increased and exceeded that of BrdU^+^ SP T cells by day 7 of continuous labeling **(** **Fig. 6E** **)**. The frequency of B220^+^ DN T cells was increased from 50 to 65% during the same period; however, it was not at the expense of SP T cells whose absolute numbers also increased [[49] and data not shown] even though they frequency decreased due to the dramatic increase in the absolute numbers of DN T cells as we previously reported [49].

In summary, our results show that DN T cells in wt mice are proliferating and dying at exceptionally high rates than SP T cells in the steady state. The high proliferation rate is restricted to DN T cells found in the gut epithelium whereas the high apoptosis rate occurred both in the gut epithelium and periphery. However, only in the periphery DN T cell apoptosis is Fas-dependent. The high sensitivity of DN T cells to Fas-mediated apoptosis in the periphery appears to be an important factor in maintaining their polarized tissue distribution and prevention of their accumulation in the periphery.

## Discussion

This study shows DN T cells in the gut epithelium were proliferating and dying at exceptionally high rates than SP T cells in wt mice. Both proliferation and apoptosis of DN T cells in the gut epithelium were regulated by Fas-independent mechanisms. Consequently, impairment of the Fas pathway had minimal impact on the overall homeostasis of DN T cells in the gut epithelium. By contrast, DN T cells in the periphery were cycling at comparable rate as SP T cells but were dying at a very high rate and hence remained a negligible component of the peripheral T cell repertoire. The Fas pathway played a non-redundant role in the apoptosis of peripheral DN T cells. Consequently, impairment of the Fas pathway reduced apoptosis of peripheral DN T cells to a baseline level similar to that of SP T cells. Under these conditions of normalized apoptosis, Sdc1^+^ B220^+^ DN T cells progressively accumulated in the periphery resulting in what is referred to as B220^+^ DN T cell lymphoproliferation. Thus, it appears that DN T cells are directly targeted and removed by Fas-mediated apoptosis from the periphery and that in the absence of this regulatory mechanism, homeostasis and tissue distribution of DN T cells become dysregulated. These results provide new insights into pathogenesis of B220^+^ DN T cell lymphoproliferation.

The current prevailing view is that B220^+^ DN T cell lymphoproliferation is caused by SP T cells that failed to undergo apoptosis and persisted as B220^+^ DN T cells after upregulation of B220 and downregulating their coreceptors to dampen autoreactivity. While we cannot formally rule out some conversion of SP into B220^+^ DN T cells, the microarray analysis indicates that the transcript profile of gld DN T cells is complex and with significant qualitative difference from the transcript profile of gld SP T cells **(** **Fig. 2** **)**. Therefore, if B220^+^ DN T cells were derived from SP T cells they must have undergone complex differentiation process that extended beyond simple loss of the coreceptor and upregulation of B220. Furthermore, while the absolute numbers of both SP and B220^+^ DN T cells progressively increased in the periphery of gld mice, accumulation of B220^+^ DN T cells occurred at a rate that significantly exceeded that of SP T cells eventually resulting in predomination of the repertoire by B220^+^ DN T cells. There is about 10-fold increase in the absolute numbers of SP T cells in lpr and gld mice by the age of 16 weeks compared to several thousand-folds increase in the numbers of B220^+^ DN T cells [49]. Consequently, the frequency of SP T cells decreased (from 95% in 6-week-old to less than 20% by the age of 16 weeks) while that of B220^+^ DN T cells increased (from less than 10% to more than 80% by the age of 16 weeks) in mutant mice. Chronically activated autoimmune T cells that are normally deleted by Fas-mediated apoptosis [50], [51] constituted most of SP T cells that persist in mutant mice as acutely activated SP T cells died normally or with little delay and without causing a lingering effect on B220^+^ DN T cells **(** **Fig. 1** **)**. Thus, many SP T cells that accumulate in mutant mice did so without losing their coreceptors.

Mathematically, it would be impossible for conversion of SP into B220^+^ DN T cells to account for B220^+^ DN T cell lymphoproliferation unless newly generated B220^+^ DN T cells were proliferating at higher rate or dying at lower rate than SP T cells in mutant mice. However, these were not the cases, as the cycling rate of SP and B220^+^ DN T cells in the periphery of gld mice were not significantly different. Furthermore, there were no survival advantages for B220^+^ DN T cells over SP T cells in the periphery of gld mice. In fact B220^+^ DN T cells chances of surviving in gld mice were not better than those of CD8 T cells and worse than those of CD4 T cells **(** **Fig. 6E** **)**. Hence, the increase in the frequency of DN T cells occurred despite a parallel increase in the absolute number of SP T cells and hence was not at the expense of SP T cells. In the light of the new data presented in this study, we propose the alternative hypothesis that autoimmune SP and B220^+^ DN T cells are independently regulated by Fas-mediated apoptosis and thus there is parallel but independent accumulation of B220^+^ DN and SP T cells in the periphery of mice with impaired Fas pathway. However, accumulation of B220^+^ DN T cells occurs at a rate that significantly exceeds that of SP T cells eventually resulting in predomination of the repertoire by B220^+^ DN T cells.

This study raises several important questions: Why DN T cells are directly targeted and eliminated from the periphery of wt mice by Fas-mediated apoptosis in the steady state. There may be a customized/specific role for DN T cells in regulating immune responses in the gut epithelium but not the periphery and hence their presence in the periphery is undesirable. Consistent with this view, B220^+^ DN T cell accumulation in the peripheral lymphoid organs is associated with systemic autoimmunity in mice with certain genetic backgrounds [17] and in children with ALPS [52], [53]. In addition, it leads to severe lymphadenopathy and splenomegaly that occasionally require surgical intervention in ALPS patients to relieve organ compression [54]. If B220^+^ DN T cells were not immediate progeny of SP T cells, what is their source? The definitive source of B220^+^ DN T cells that accumulate in the periphery is currently unknown and beyond the scope of this study. However, it will be important in the future to determine whether peripheral B220^+^ DN T cells had previously resided in the gut epithelium or came directly from the thymus. The former possibility is supported by our data and by previous studies showing existence of lpr-like B220^+^ DN T cells in the appendix [34] and genital tract [55] of wild type mice. New experimental systems are required to approach this issue, *however*.

In summary, this study shows that DN T cells are proliferating and dying at exceptionally high rates than SP T cells in the steady state in wild type mice. The high proliferation rate is restricted to DN T cells found in the gut epithelium whereas the high apoptosis rate occurred both in the gut epithelium and peripheral lymphoid tissues of wild type mice. However, only in the periphery that DN T cell apoptosis is Fas-dependent. The high sensitivity of DN T cells to Fas-mediated apoptosis in the periphery appears to be an important factor in regulating their tissue distribution and homeostasis. These results have important implications for understanding pathogenesis of B220^+^ DN T cell lymphoproliferation that occurs in mice and children with genetically impaired Fas pathway. Future studies should investigate mechanisms underlying rapid proliferation of DN T cells in the gut epithelium and how their presence in the periphery influences peripheral tolerance and autoimmunity.

## Materials and Methods

### Mice

C3H mice carrying homozygous loss-of-function gld and lpr mutations of FasL and Fas, respectively, C3H and NOD wild type mice were purchased from Jackson Laboratory. NOD-gld/gld mice are previously described [49]. All mice were bred and maintained at the Animal Care Facility of the Johns Hopkins School of Medicine.

### Isolation of peripheral T cells

DN T cells were isolated by negative selection by using Dynabeads (Dynal®, Lake Success, NY) as previously described [42]. Briefly, single cell suspensions from inguinal and axillary lymph nodes were incubated with a cocktail of biotin-conjugated rat mAbs specific for murine CD4, CD8, I-E^k^, and CD16/CD32 surface molecules. Streptavidin-conjugated beads were used to capture and remove mAb-coated cells. Single positive (CD4 and CD8) Sp T cells were isolated by the same technique using a cocktail of biotin-conjugated rat mAbs specific for murine I-E^k^, CD16/CD32 and B220 surface molecules. For isolation of CD4 T cells, anti-CD8 antibody was added to the cocktail of I-E^k^, and CD16/CD32 and B220 biotin-conjugated rat mAbs. Purity of isolated T cells was by assessed by staining samples with APC-TCRβ, PerCP-CD4, PerCP-CD8, and Fitc-B220 specific mAbs or with APC-TCRβ, PerCP-CD4, PE-CD8, Fitc-B220 mAbs followed by analysis with FACSCalibur. The purity of isolated DN T cells, SP T cells used in these studies was more than 95%.

### Isolation of IEL

This procedure is performed as described by Davies and Parrott [56] with slight modifications. Intestines were dissected from their mesentery, and the Peyer's patches and lymphoid aggregates were removed. Small or large intestine tissues from two to three mice in each group were pooled together to increase cell yield. Intestines were cut longitudinally into 0.2- to 0.5-cm pieces and washed with HBSS. The epithelial layer was removed by incubating in HBSS containing 5 mM dithiothreitol for 10 min while stirring to remove mucus, and then incubated at 37°C on a shaker three times for 20 min at 250 rpm in HBSS (without Ca^2+^ and Mg^2+^) containing 1 mM EDTA. After washing, IEL and enterocytes were separated on a discontinuous density gradient (25%, 40%, and 75%). Cells that layered between the 40% and 75% fractions were collected as IEL. Viability of isolated cells was determined by trypan blue exclusion. H597 antibody that recognizes Cβ chain of the TCR was used to identify TCRαβ-expressing IEL.

### Staining and flow cytometry

Cells were stained at 4°C with the following antibodies in PBS with 5% FCS: FITC-, PE, PerCP, and APC-conjugated specific mAbs (BD Pharmingen or eBioscience) unless otherwise stated. The antibodies used include: anti-TCRβ, anti-CD4, anti-CD8α, anti-CD2, anti-B220, anti-CD5, anti-Fas, anti-Thy1, anti-CD24, ant-CD62L, anti-sdc1, anti-Vβ13, anti-Vβ14, and anti-Vβ6. In many instances, CD4 and CD8 T cells were analyzed together in one subset referred to as single positive (SP) T cells by simultaneously staining samples with PerCP-conjugated anti-CD4 and anti-CD8α mAbs. Inclusion of SP T cells in one subset allowed us to compare their properties directly with those of DN T cells in dot plots. Data were collected using a FACSCalibur flow cytometer and analyzed by the CELL Quest software or FCS Express. Gating of positively stained populations was determined using isotype-matched antibodies.

### Analysis of expansion and deletion of SEB-reactive T cells

C3H-gld/gld or C3H-wt mice at 14 weeks of age were injected i.p. with staphylococcal enterotoxin B (SEB) obtained from Toxin Technology. Expansion and deletion of SEB-reactive (Vβ8) and non-reactive (Vβ6) T cells were determined by monitoring the frequencies of each subset in peripheral blood over a period of 12 days.

### Bromodeoxyuridine (BrdU) analysis

For proliferation experiments, mice were injected i.p. with 2 mg of BrdU in PBS twice within a 24-h period, followed by tissue collection and analysis of T cell subsets for BrdU incorporation using the BrdU flow kit protocol (BD Pharmingen). For turnover analysis, mice were injected with 2 mg/ml of BrdU i.p. and then maintained on daily-prepared drinking water containing BrdU at 0.8 mg/ml, as previously described [48]. BrdU given at 0.8 mg/ml could be administered for several weeks without apparent cytotoxicity [57], [58]. In brief, cells were surface-stained with APC-TCR, PerCP-CD4/CD8, and PE-sdc1 antibodies, fixed, permeabilized, re-fixed and treated with DNase. Cells were stained with BrdU-specific mAb and analyzed using FACSCalibur.

### Analysis of Fas-mediated death in vivo

FasL-neutralizing mAb MFL4 was previously described [45]. Mice were injected intraperitoneally (0.5 mg/mouse, twice within 24-h period) with MFL4 or hamster IgG. The percentages of apoptotic T cells among T cells isolated from the gut epithelium and the periphery were determined using annexin V apoptosis detection kit from BD Pharmingen.

### Analysis of sensitivity to Fas-mediated death in vitro

Gld DN and CD4 T cells (>95% pure) were isolated by negative selection with Dynal beads as described above. Each cell type was incubated (1×10^5^ cells/well) in triplicate with 1 ng/ml of recombinant human FasL (rhFasL, Alexis Inc) in the presence of 1 µg/ml enhancer protein for 16 h at 37°C. At the end of incubation, cells were stained with APC-TCR and PerCP-CD4 antibodies and analyzed for annexin V binding as above.

### DNA microarray analysis

Total RNA was isolated from highly purified DN or SP T cells (>99%) by using the RNeasy Mini Kit (QIAGEN). Biotinylated cRNA was fragmented and hybridized to the Affymetrix murine genome GeneChip array MOE430 set, according to manufacturer's instructions. To assess the quality of the hybridization, GeneChip image, and comparison among chips, we confirmed the following parameters: scaling factor values within comparable range (between 0.7 and 1.4); low background values (between 51 and 70); high percentage of present calls (between 36 and 50); consistent 3′/5′ ratios of GAPDH as representatives of housekeeping genes; and presence or absence of Bio B and C as internal spike controls [49]. Statistical analysis of microarray data was performed as recently described by our group [49].

### Real-time quantitative PCR

To measure mRNA transcripts, DN and SP T cells were isolated from the lymph nodes of C3H-gld/gld mice as described above. Total RNA isolation, first strand cDNA synthesis, and real-time reactions were performed as previously described [49].
